# Cytochrome P450 enzymes as modulators of oncogenic signaling via the Wnt/β-catenin signaling pathway

**DOI:** 10.1007/s00204-025-04295-7

**Published:** 2026-01-17

**Authors:** Albert Braeuning

**Affiliations:** Department Chemical and Product Safety, German Federal Institute for Risk Assessment, Max-Dohrn-Str. 8-10, 10589 Berlin, Germany

**Keywords:** Beta-catenin, Carcinogenesis, CYP, Liver, Nuclear receptors, Xenobiotics

## Abstract

Enzymes from the cytochrome P450 (CYP) superfamily, especially from families CYP1, CYP2, and CYP3, play a decisive role in phase I of drug and xenobiotic metabolism in mammalian organisms. The enzymes are responsible for metabolic conversion and detoxification of a plethora of foreign molecules. Metabolic conversion of pro-carcinogenic compounds links CYP enzyme activities to cancer development, while in addition oncogenic pathways have been shown to regulate the expression of CYP genes, together with the well-known regulation by nuclear receptors acting as ligand-activated transcription factors triggered by exposure to xenobiotics. Specifically, the Wnt/β-catenin signaling pathway is among the recently established transcriptional regulators of CYP enzymes. β-Catenin is well-known as a key player in organism development and, when aberrantly activated, a major oncogenic driver of carcinogenesis. While the latter phenomena are rather well-described, new evidence suggests that CYP enzymes themselves may, under certain conditions, also affect the activity of the β-catenin pathway and thereby could impact on carcinogenesis in a way different from toxifying or detoxifying foreign compounds. This review focuses on the currently available knowledge about the regulation of β-catenin-dependent signaling by CYP enzymes. The synopsis of data reveals the possibility of a previously undervalued role of CYPs in the regulation of Wnt/β-catenin signaling, and possible molecular mechanisms are highlighted.

## Introduction

Cytochrome P450 enzymes constitute a large and evolutionary conserved superfamily of proteins carrying out different functions in animals, plants, and fungi. A huge and still growing number of members of the CYP superfamily have been identified in a plethora of different species (Nelson 1998, 1999). In mammalian organisms, CYPs from the families 1, 2, and 3 fulfill important tasks in the metabolism of drugs and xenobiotics, exhibiting broad and overlapping substrate specificities, which enable them to metabolize the vast majority of foreign compounds entering the body, thus substantially contributing to the organism’s detoxification processes. CYPs are expressed in various organs, often in tissues providing some kind of barrier function. Highest expression of many CYP enzymes from families 1–3 is observed in the liver, the central organ of detoxification. By contrast, CYPs from families with higher family numbers mostly catalyze metabolic reactions related to endogenous metabolism. For a comprehensive review of CYP isoforms involved in human drug metabolism, see Zanger and Schwab (2013).

Regulation of hepatic expression of many important CYPs from families 1–3 is regulated by ligand-activated transcription factors from the nuclear receptor family, for example the constitutive androstane receptor (CAR) or the pregnane-X-receptor (PXR), in response to exposure to xenobiotics activating these receptors (Honkakoski and Negishi 2000; Pelkonen et al. 2008). Also, additional players such as transcription factors from the hepatocyte nuclear factor family (e.g., see (Liu and Gonzalez 1995), or hormone-related signaling processes (e.g., see Liddle et al. 1998) have been identified as regulators of CYP expression. About two decades ago, it has been discovered that also the canonical Wnt/β-catenin signaling pathway is a regulator of hepatic CYP expression in liver cells (Loeppen et al. 2005). Subsequent research identified β-catenin as a master regulator not only of CYP expression in the liver, but also as a decisive factor of hepatic zonation, affecting the magnitude as well as the localization and zonation of gene expression (Braeuning and Schwarz 2010; Carson and Nejak-Bowen 2025; Goel et al. 2022; Hailfinger et al. 2006; Yang et al. 2014).

**Fig. 1:**
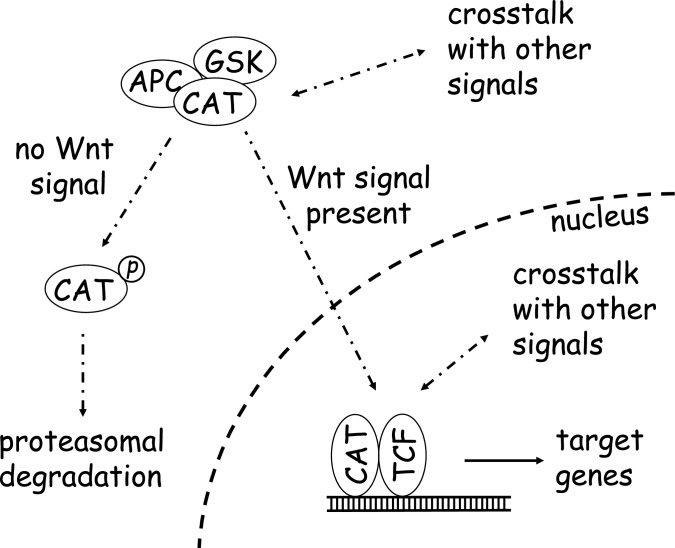
Schematic representation of the canonical Wnt/β-catenin signaling pathway. In the absence of an activating Wnt signal, β-catenin (CAT) is phosphorylated by a cytosolic multi-protein complex including the adenomatous polyposis coli (APC) and glycogen synthase kinase 3β (GSK) proteins, followed by ubiquitinylation and proteasomal degradation of the phosphorylated β-catenin. Wnt signals inhibit β-catenin phosphorylation and degradation, so that the protein can accumulate and translocate to the nucleus, where it is an activator of transcription of target genes together with transcription factors from the T-cell factor (TCF)/lymphoid enhancer factor family. Crosstalk with other signaling pathways can occur at the cytosolic as well as nuclear levels

The Wnt/β-catenin pathway pathway has multiple functions in the body and has been traditionally recognized mainly as an important signaling cascade regulating early developmental processes, as well as an oncogenic driver of carcinogenesis in different organs (Carson and Nejak-Bowen 2025; Liu et al. 2022; Logan and Nusse 2004; Matsumoto and Kikuchi 2024): Wnt molecules are the physiological activators of the pathway. Their binding to Frizzled Receptors modulates an intracellular machinery responsible for regulating the phosphorylation, ubiquitinylation and subsequent proteasomal degradation of β-catenin. While in the absence of a Wnt signal, β-catenin is continuously phosphorylated and degraded, Wnt activity interrupts this process and induces cellular accumulation of β-catenin, which will then be able to translocate into the nucleus to control the expression of its target genes following binding to T-cell factor/lymphoid enhancing factor (TCF/LEF) transcription factors. The pathway is schematically delineated in Fig. 1.

Quite some literature has been published about the regulation of CYPs by β-catenin, so that regulation of xenobiotic-metabolizing CYPs by the β-catenin pathway can now be regarded as established (see citations above). Molecular mechanisms have been identified including β-catenin/TCF binding to CYP gene promoters, interplay between HNFs and β-catenin in the regulation of transcription, as well as cooperation of β-catenin and nuclear receptors in xenobiotic-activated CYP expression (Braeuning et al. 2011; Giera et al. 2010; Groll et al. 2016; Schulthess et al. 2015).

While the main task of CYP enzymes from families 1–3 appears to be defense against exposure to foreign compounds, it should not be overlooked that additional functions of these CYPs may exist that have not been researched in depth so far. Many interactions between different pathways or signaling cascades are not unidirectional but encompass mutual effects of the two pathways on each other. Thus, it makes sense to assume that the Wnt/β-catenin is not only a regulator of CYP enzymes, but that some CYP enzymes may, to a certain extent, also be able to modulate the activity of β-catenin-dependent signaling. This review focuses on the effects of CYPs from families 1–3 on Wnt/β-catenin signaling, identifying published direct and indirect interactions, and highlighting molecular mechanisms of these interactions. This review does thereby not focus on effects of the β-catenin signaling pathway on CYP expression, and also not on the interplay of CYP-regulating nuclear receptors with β-catenin. Furthermore, with respect to the potential role of CYP enzymes in tumorigenesis, the interplay of CYPs with anticancer drugs is not in the focus of this work, but have been reviewed elsewhere recently (Lee et al. 2025).

### CYP family 1

CYP family 1 members are mainly known for their role in the metabolism of planar hydrophobic molecules, such as polycyclic aromatic hydrocarbons or certain polychlorinated biphenyls (Abel and Haarmann-Stemmann 2010; Haarmann-Stemmann and Abel 2006). The aryl hydrocarbon receptor (AHR) is known as a xenobiotic-activated master regulator of CYPs, especially from the CYP1A and CYP1B subfamilies (Tompkins and Wallace 2007). Several studies have been published linking the AHR and its target CYPs to the activity of the Wnt/β-catenin signaling pathway: for example, the AHR is a transcriptional target of the β-catenin pathway (Chesire et al. 2004), and β-catenin cooperates with the AHR in the regulation of *CYP1A1* expression and regulation of zonal gene expression in healthy liver (Braeuning et al. 2011; Schulthess et al. 2015). Independent of β-catenin, the AHR has been identified as involved in carcinogenesis, activated by either exogenous or endogenous ligands, with pro- as well as anti-carcinogenic effects, depending on the individual context of ligand and tissue (Murray et al. 2014). An interesting interplay of AHR- and β-catenin-driven pathways, independent of CYP regulation, has been described (Grishanova et al. 2023; Schneider et al. 2014). While the above phenomena can be considered rather well-established, linking activity of β-catenin to the regulation of AHR target CYPs, not much data are available characterizing possible links into the reverse direction, i.e., from CYP1 family members to the Wnt/β-catenin pathway.

Most work into this direction has been performed on CYP1B1. High expression of CYP1B1 is, for example, observed in adenoma and carcinoma of the colon (e.g., see Chang et al. 2005; Liu et al. 2025a) Z10/16. Of note, immunohistochemical assessment of the abundance of CYP1B1 and β-catenin in combination has been suggested as a marker for colon carcinoma identification (Chang et al. 2005). This obvious connection could, in principle, be related (i) to independent mechanisms of both proteins acting on the carcinogenic process or being influenced by the carcinogenic process, (ii) to the regulation of CYP1B1 by active β-catenin, or (iii) due to effects of CYP1B1 on the Wnt/β-catenin pathway. Interestingly, there is published evidence that the latter scenario plays a role: overexpression of CYP1B1 in vitro enhances proliferation and epithelial-to-mesenchymal transition (EMT) in human colon carcinoma cells, whereas CYP1B1 knockdown produced inverse effects (Liu et al. 2025a). Similarly, it has been observed that in MCF-7 and MCF-10A breast cancer cells overexpression of CYP1B1 leads to elevated proliferation, EMT and migration (Kwon et al. 2016). In the latter paper, the authors showed that CYP1B1 regulates β-catenin via an upregulation of the expression of *CTNNB1*, by a mechanism involving the transcription factor SP1 (Kwon et al. 2016). Of note, 4-hydroxyestradiol, a major estradiol metabolite generated by CYP1B1, induced similar effects as CYP1B1 overexpression, suggesting that this metabolite might mediate the effects of CYP1B1 on cellular proliferation (Kwon et al. 2016). In another publication evidence has been presented that in HeLa cells, CYP1B1 inhibits ISGylation by controlling the expression of the Herc5, a ligase for ISGylation, and that Herc5 and β-catenin directly interact (Park et al. 2017). Therefore, CYP1B1 appears to contribute to β-catenin stabilization by diminishing β-catenin proteasomal degradation through inhibition of β-catenin ISGylation (Park et al. 2017). The fact that tetramethoxystilbene, an inhibitor of CYP1B1 activity, decreased proliferation and related effects in the latter study (Park et al. 2017), again points towards a crucial role of CYP1B1-generated metabolites in mediating the proliferative effects of this CYP enzyme. Additional evidence for a proliferative role of CYP1B1 comes from a paper showing accelerated wound healing by CYP1B1 via activation of the Wnt/β-catenin pathway (Zuo et al. 2024). However, it should be noted that there are also data available not showing effects of CYP1B1 on the β-catenin pathway, for example in brain vascular endothelial cells, where CYP1B1 knockdown did not affect β-catenin levels, GSK3β or Wnt3a (Chen et al. 2024).

Effects of other CYP family 1 members on the β-catenin pathway have also been subject of research, even though less publications are available than for CYP1B1. Studies with breast cancer stem cells have shown that activation or inactivation of AHR, and thus subsequent induction of CYP1A1, increased or decreased, respectively, cancer stem cell marker and mammosphere formation (Al-Dhfyan et al. 2017). Inactivation of the AHR sensitized cells against doxorubicin toxicity (Al-Dhfyan et al. 2017). Activation of AHR was linked to increased nuclear β-catenin and elevated levels of cyclin D1, a downstream target of β-catenin signaling and key proliferation stimulator. Experiments demonstrating that a knockdown of CYP1A1 reversed the effects of AHR modulation demonstrate that the observed effects are indeed mediated by CYP1A1, and further evidence was presented that the PTEN/AKT pathway is involved in the regulation of β-catenin signaling by CYP1A1 in breast cancer cells (Al-Dhfyan et al. 2017). Of note, a complex crosstalk of AKT and β-catenin signaling has been described previously, for example by Fleming-de-Moraes et al. (2022) and Jere et al. (2023), including the inhibition of GSK3β and thus activation of β-catenin by AKT activation. It is important to state that the abovementioned effects of CYP1A1 are not dependent on the AHR, as modulation of the AHR alone may on its own alter β-catenin activity, even though not in a way that is within the scope of this work, as for example reviewed in Grishanova et al. (2023) and in Schneider et al. (2014). The CYP isoform 1A2 has also been identified as a player in tumorigenesis in human hepatocellular carcinoma (HCC) cell lines, as it was shown to act as an antagonist of HGF/MET signaling by binding to HIF1α, a transcriptional activator of MET (Yu et al. 2021). As MET activation induces β-catenin signaling in hepatocytes (Monga et al. 2002), one might expect that modulation of MET signaling by CYP1A2 subsequently impacts β-catenin signaling.

### CYP family 2

The CYP family 2 members are diverse in their regulation and substrate specificities. Important transcriptional regulators include especially the constitutive androstane receptor CAR and the pregnane-X-receptor PXR, regulating for example CYP2 from subfamilies 2B and 2C in response to xenobiotic exposure (Honkakoski and Negishi 2000; Kobayashi et al. 2015). As for AHR-regulated CYPs from the CYP1 family, expression of different CAR- and/or PXR-dependently regulated CYP2 and CYP3 isoforms has been reported to be co-regulated by β-catenin signaling, and also similar to CYP1 isoforms, β-catenin is a crucial player in determining zone-specific expression of those CYPs in the liver (Braeuning et al. 2009; Braeuning and Schwarz 2010); see also additional citations above). Activation of CAR and also an interplay of signaling through CAR and β-catenin has been reported to play a role in hepatocarcinogenesis (Braeuning and Pavek 2020; Braeuning et al. 2010; Dong et al. 2015; Rignall et al. 2011). Human relevance of tumor-promoting effects mediated by CAR, however, has been questioned due to species differences between rodents and humans (Elcombe et al. 2014). While thus, on the one hand, a considerable amount of data is present demonstrating effects of β-catenin signaling on CYP2 and CYP3 family members, data on effects of CYPs on the β-catenin pathway are scarce.

CYP2A6 has been implied in the regulation of β-catenin-dependent signaling in hepatocarcinoma cells: overexpression of CYP2A6, normally decreased in HCC patient samples, reduced proliferation, migration and invasion in vitro, as well as diminished tumorigenicity and metastasis in vivo in xenograft experiments with nude mice (Liu et al. 2025b). The authors demonstrated that this effect was independent of the metabolic function of CYP2A6; instead, a mechanism was revealed showing binding of CYP2A6 to the tyrosine kinase SRC, thus facilitates β-catenin proteasomal degradation via altered phosphorylation levels of the Dvl2, a crucial player in Wnt-dependent activation of β-catenin signaling (Liu et al. 2025b). Also, CYP2E1, traditionally known for its metabolism of small xenobiotics such as ethanol or acetaminophen (Chen et al. 2019) and a direct target of the β-catenin pathway (Gerbal-Chaloin et al. 2014; Groll et al. 2016), has been proposed to play a role in regulating the Wnt/β-catenin pathway: CYP2E1 overexpression was found to increase migration and invasion in hepatocarcinoma cells, while silencing of the enzyme yielded opposite results (Ishteyaque et al. 2025). This occurred alongside with an activation of β-catenin signaling and reactive oxygen species (Ishteyaque et al. 2025). Of note, reactive oxygen species have been described to transiently activate the Wnt/β-catenin pathway during developmental processes (Haack et al. 2015). CYPs, especially CYP2E1, are a potential source of reactive oxygen species (Harjumaki et al. 2021), thus potentially affecting β-catenin signaling via those reactive molecules. By contrast, the authors of another study published evidence that CYP2E1 acts antagonistically on β-catenin-dependent tumorigenesis, as CYP2E1 overexpression diminished β-catenin signaling activity in hepatocarcinoma cells (Zhu et al. 2022). This phenomenon was proposed to be linked to decreased Dvl2 expression and CYP2E1-promoted ubiquitin-mediated Dvl2 degradation by a mechanism involving stabilization of Dvl2 binding to a ubiquitin ligase, and the latter effect was downstream of CYP2E1-induced accumulation of reactive oxygen species (Zhu et al. 2022). Accordingly, the authors observed, as others before, downregulation of CYP2E1 in hepatocellular carcinoma samples (Zhu et al. 2022).

CYP2J2 is a CYP isoform that is not frequently in the focus of research. Nonetheless, there is one publication available linking CYP2J2 and its upregulation in human tumor samples to a pro-carcinogenic role of this CYP enzyme (Jiang et al. 2005): CYP2J2 overexpression in vitro induced and CYP2J2 knockdown diminished proliferation of cancer cells, and CYP2J2 protected cells from tumor necrosis factor (TNF)α-induced apoptosis (Jiang et al. 2005). The possibility to antagonize the effects of CYP2J2 by an epoxygenase inhibitor links the effects of CYP2J2 to its catalytic activity (Jiang et al. 2005). The authors ascribe the observed phenomena to the function of CYP2J2 as an arachidonic acid-metabolizing enzyme, generating epoxyeicosatrienoic acids which in turn affect the mitogen-activated protein kinase (MAPK) and the PI3K/AKT signaling systems (Jiang et al. 2005). As a consequence of the interlinking between β-catenin- and AKT-dependent signaling mentioned before (Fleming-de-Moraes et al. 2022; Jere et al. 2023), as well as of the multifaceted crosstalk between MAPK and β-catenin signaling (e.g., reviewed in Zeller et al. 2013), CYP2J2 could exert at least some of its procarcinogenic effects via modulation of the β-catenin pathway. More evidence for a role of CYP-generated arachidonic acid-derived metabolites in the regulation of proliferation by MAPK and SRC signaling has been produced in a study with renal epithelial cells, even though the link to specific CYP isoforms is not entirely elucidated in this work (Chen et al. 1998).

More publications than for CYP2J2 are able for CYP2S1 with regards to its ability to affect proliferation, oncogenic signaling and tumorigenesis, and again there is a strong link to metabolites of arachidonic acid generated by this CYP isoform. With respect to the aforementioned CYP2S1, depletion by RNAi promoted cell proliferation through increasing levels of prostaglandin E2 (PGE2) in HCT116 carcinoma cells (Yang et al. 2015). Similar findings were also observed in another study with colorectal cancer cells (Du et al. 2025). PGE2, in turn, reduced β-catenin phosphorylation, thus activating β-catenin-dependent transcription (Yang et al. 2015). Accordingly, CYP2S1 knockdown induced tumor growth in a xenograft model in vivo (Yang et al. 2015). Even though not showing a direct relation to β-catenin signaling, experiments showing that siRNA against CYP2S1 leads to higher proliferation and migration of a bronchial epithelial cell line further add to the overall evidence of the above (Madanayake et al. 2012). This was attributed by the authors to the role of CYP2S1 in prostaglandin metabolism (Madanayake et al. 2012). Of note, addition of PGE2 to the cultures was able to enhance cell proliferation in that study, but did not promote migration, suggesting that modulation of PGE2 levels might not be the only cause of CYP2S1 effects on cancer-relevant processes. More in vivo evidence for a role of CYP2S1 in carcinogenesis comes from a study with APC^Min/+^ mice bearing also a knockout of *Cyp2s1*: Here, the knockout of *Cyp2s1* accelerated tumor growth and progression (Du et al. 2025). It was also observed that in *Cyp2s1* knockout mice, increased translocation of β-catenin into the nucleus and induction of subsequent signaling occurs (Du et al. 2025). Inversely, P53 stabilization by oxaliplatin upregulated CYP2S1 and reduced β-catenin signaling in HCT116 cells (Yang et al. 2016). In one single report, the role of CYP2D6 has been addressed, revealing that overexpression of the enzyme decreased growth of hepatocarcinoma cells in vitro (Oguro et al. 2011); however, without linking the enzyme to modulation of the β-catenin pathway.

### CYP family 3

Among the CYP family 3 is CYP3A4, the probably most important and abundant CYP in humans, with a remarkably broad substrate specificity, making it a crucial player in the metabolism of numerous drugs and xenobiotics. PXR is known as the key regulator of CYP3A4 (Honkakoski and Negishi 2000; Pelkonen et al. 2008), and there is also evidence that CYP3A isoforms are co-regulated in expression by the Wnt/β-catenin signaling pathway (Braeuning et al. 2009; Braeuning and Schwarz 2020).

Overexpression of CYP3A4 in Hep3B hepatocarcinoma cells, on the one hand, induced cell proliferation, while, on the other hand, an azole-type inhibitor of CYP3A4 reduced proliferation (Oguro et al. 2011). CYP3A4 is capable of producing epoxyeicosatrienoic acids (EETs) from arachidonic acid (Oguro et al. 2011). The observation that an EET receptor antagonist was able to inhibit effects of CYP3A4 on cell growth demonstrates that EET formation plays an essential role in mediating the effect of CYP3A4 on tumor cell growth (Oguro et al. 2011). Similarly, the effect was antagonizable by a PI3K inhibitor, demonstrating involvement of the latter kinase (Oguro et al. 2011). In another study, CYP3A4-mediated proliferation in MCF7 breast cancer cells has been attributed to EET formation and subsequent activation of STAT-dependent signaling (Mitra et al. 2011). Even though clear evidence for a role of β-catenin signaling in this process is still elusive, crosstalk between Wnt/β-catenin-, STAT-, and PI3K-dependent signaling pathways has been observed (Zhao et al. 2020), making effects on β-catenin signaling a possible scenario. Downregulation of CYP3A5 has been observed in HCC, and low expression of CYP3A5 was associated with increased malignancy (Jiang et al. 2015). Analyses in hepatocarcinoma cells revealed that CYP3A5 reduces migration and invasion by diminishing matrix metalloprotease activities via a mechanism inhibiting AKT signaling (Jiang et al. 2015). Given the crosstalk of signaling pathways as already delineated above, it appears possible that this mechanism also involves modulation of Wnt/β-catenin signaling.

## Summary and conclusion

**Fig. 2:**
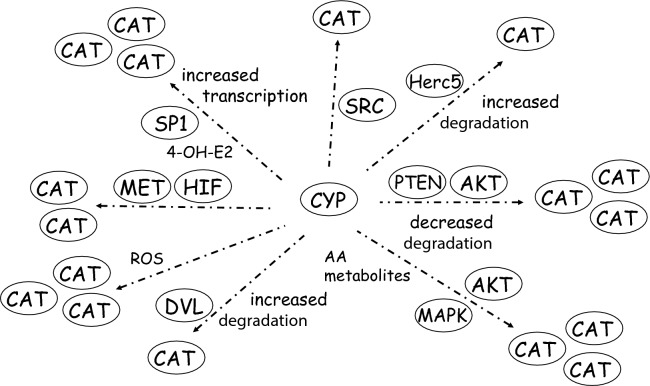
Synposis of proposed mechanisms by which CYP enzymes affect the activity of the Wnt/β-catenin signaling pathway. For a detailed description of mechanisms how CYP enzymes affect activity of β-catenin (CAT), please refer to the main text

In summary, the synopsis of available data on the regulation of Wnt/β-catenin-dependent signaling by CYP enzymes reveals that members of the CYP enzyme family may contribute to the modulation of the β-catenin pathway to a degree that has previously been underrated by most researchers in the field. A synopsis of proposed mechanisms is presented in Fig. 2, showing that multiple molecular mechanisms appear to be relevant, involving effects of endogenous CYP metabolites, direct protein–protein binding, altered protein stability of β-catenin, and various phosphorylation-dependent signaling pathways. This way, CYP expression in tumors cells may contribute to oncogenic processes. Historically, the expression of CYPs in pre-cancerous lesions has been perceived as evidence for a toxin resistance phenotype of these lesions, with the CYP expression allowing them to get insensitive against toxicological insults from the outside world. In addition to this mechanism, the new data on the modulation of the Wnt/β-catenin signaling pathway by CYP enzymes also make it tempting to speculate that at least a part of the positive effect of CYP expression in such early tumor cells might be related to the β-catenin activation and subsequent increased proliferation, and not only to protection from toxic injury by foreign compounds. The observed effects of CYPs on β-catenin are not restricted to a single individual CYP enzyme, but have been described for CYPs from different families, demonstrating a broader relevance of the topic. To some extent, molecular mechanisms of this interaction have been unraveled, and relevance of the CYP-dependent effects on β-catenin signaling for tumorigenesis has been demonstrated. Further research in the field, however, will be necessary to deepen our understanding of the exact molecular roles of CYP enzymes and their interaction with the β-catenin pathway in the development of neoplastic diseases. Novel questions arise with increasing knowledge of CYP effects on oncogenic signaling: which tumor entities in humans are significantly affected and/or influenced in their development by the above mechanisms? Can a potential contribution of CYPs to cancer in humans via the described mechanisms be quantified, and which possibilities exist to modulate the interaction from a pharmacological perspective? Future studies will help to elucidate the field more and close existing data and knowledge gaps.
